# Trimester-Specific Dietary Intakes in a Sample of French-Canadian Pregnant Women in Comparison with National Nutritional Guidelines

**DOI:** 10.3390/nu10060768

**Published:** 2018-06-14

**Authors:** Claudia Savard, Simone Lemieux, S. John Weisnagel, Bénédicte Fontaine-Bisson, Claudia Gagnon, Julie Robitaille, Anne-Sophie Morisset

**Affiliations:** 1 School of Nutrition, Laval University, Québec City, QC G1V 0A6, Canada; claudia.savard.4@ulaval.ca (C.S.); simone.lemieux@fsaa.ulaval.ca (S.L.); julie.robitaille@fsaa.ulaval.ca (J.R.); 2 Endocrinology and Nephrology Unit, CHU de Québec-Université Laval Research Center, Québec City, QC G1V 4G2, Canada; john.weisnagel@crchudequebec.ulaval.ca (S.J.W.); claudia.gagnon@crchudequebec.ulaval.ca (C.G.); 3 Institute of Nutrition and Functional Foods, Laval University, Québec City, QC G1V 0A6, Canada; 4 Department of Medicine, Laval University, Québec City, QC G1V 0A6, Canada; 5 School of Nutrition Sciences, University of Ottawa, Ottawa, ON K1N 6N5, Canada; bfontain@uottawa.ca; 6 Institut du Savoir Montfort, Montfort Hospital, Ottawa, ON K1K 0T2, Canada

**Keywords:** pregnancy, dietary intakes, energy intakes, supplements, dietary reference intakes (DRIs)

## Abstract

Diet during pregnancy greatly impacts health outcomes. This study aims to measure changes in dietary intakes throughout trimesters and to assess pregnant women’s dietary intakes in comparison with current Canadian nutritional recommendations. Seventy-nine pregnant women were recruited and completed, within each trimester, three Web-based 24-h dietary recalls and one Web questionnaire on supplement use. Dietary intakes from food, with and without supplements, were compared to nutritional recommendations throughout pregnancy. Energy and macronutrient intakes remained stable throughout pregnancy. A majority of women exceeded their energy and protein requirements in the first trimester, and fat intakes as a percentage of energy intakes were above recommendations for more than half of the women in all trimesters. Supplement use increased dietary intakes of most vitamins and minerals, but 20% of women still had inadequate total vitamin D intakes and most women had excessive folic acid intakes. This study showed that pregnant women did not increase their energy intakes throughout pregnancy as recommended. Furthermore, although prenatal supplementation reduces the risk of inadequate intake for most micronutrients, there is still a risk of excessive folic acid and insufficient vitamin D intake, which needs further investigation.

## 1. Introduction

Pregnancy is a critical period during which the pregnant woman’s diet must provide enough nutrients to ensure optimal fetal development as well as to sustain the mother’s physiological needs. In fact, in addition to the metabolic demand associated with the fetus’ growth, rises in blood volume, extracellular liquids, adipose tissue, and placental weight all lead to an increase in the mother’s dietary requirements [1,2]. Consequently, as recommended by the Institute of Medicine and Health Canada, daily pre-pregnancy energy intakes should be increased by 340 and 452 kcal in the second and third trimesters, respectively, in order to create a positive energy balance [3,4]. Likewise, pregnant women should increase their protein intakes in the second and third trimesters, but no specific recommendation exists for carbohydrates and fats during pregnancy [3].

Higher energy intakes should allow pregnant women to meet their higher essential fatty acid, dietary fiber, folic acid, iron, vitamin D, calcium, vitamin B_12_, and vitamin C requirements [3,4]. However, previous research highlighted various dietary inadequacies, namely folate, iron, vitamin B_12_, and vitamin D insufficiencies [5,6,7] thus suggesting that pregnant women may have difficulty meeting their higher micronutrient requirements through diet alone [5]. Moreover, since inadequate folate and iron status during pregnancy has been associated with numerous adverse health outcomes [8,9,10], Health Canada recommends that pregnant women should take, on a daily basis, a multivitamin that contains at least 400 µg of folic acid and 16–20 mg of iron [4]. There are currently no specific recommendations in terms of supplementation for other micronutrients. The use of a multivitamin combined with the increase in total energy intakes is probably sufficient to allow pregnant women to fill other micronutrient requirements [11].

Dietary intakes should be examined throughout pregnancy in order to detect potential excesses or deficiencies in macro- and micronutrients associated with adverse pregnancy outcomes [12,13,14]. However, to date, few studies have assessed pregnant women’s dietary intakes by considering both food and supplement sources, and even fewer have done so in each trimester of pregnancy [15,16,17,18,19,20]. To our knowledge, no study assessed trimester-specific adequacy to current nutritional Canadian recommendations. This study aimed to: (1) measure changes in energy and macronutrient intakes across trimesters; and (2) assess pregnant women’s dietary intakes in comparison with current Canadian nutritional recommendations.

## 2. Materials and Methods

### 2.1. Study Population

Eighty-six (86) pregnant women recruited from April 2016 to May 2017 at the CHU de Québec—Université Laval (Québec City, QC, Canada) were included in the ANGE (*Apports Nutritionnels durant la GrossessE*) project. Women younger than 18 years old and with a gestational age greater than 11 weeks of pregnancy at the time of recruitment were excluded. Women with a previously diagnosed severe medical condition (i.e., type 1 or type 2 diabetes, renal disease, inflammatory and autoimmune disorders) were also excluded. Our final sample included 79 women for whom we have nutritional data in all trimesters. The ANGE project was approved by the CHU de Québec—Université Laval Research Center’s Ethics Committee and participants gave their informed written consent at their first visit to the research center.

### 2.2. The Automated Web-Based 24-h Recall (R24W)

In the first (range: 8.4–14.0 weeks), second (range: 19.3–28.3 weeks), and third (range: 31.9–37.7 weeks) trimesters of pregnancy, each participant was asked to complete a total of three Web-based 24-h dietary recalls, using the R24W (Rappel de 24h Web; 24h dietary recall) platform, on two weekdays and one weekend day (total of nine dietary recalls throughout pregnancy). The development of the R24W has been previously described [21]. Briefly, the R24W uses a sequence of questions inspired by the United States Department of Agriculture (USDA) Automated Multiple Pass Method (AMPM) [22]. The application sends automatic emails on randomly chosen dates to remind the participants to complete the recall. Participants were required to watch a mandatory tutorial video prior to their first recall. The database includes 2865 food items that are linked to the Canadian Nutrient File [23] to enable automatic extraction of nutrient values. Participants can report an unlimited number of meals and snacks for a 24-h period. Pictures depicting multiple portion sizes with corresponding units and/or volume are available for more than 80% of all food items. After selecting a food item, participants must choose the picture that best represents the amount of food eaten. In addition, systematic questions are asked about frequently forgotten food items including toppings, condiments, fats, snacks, and drinks. The R24W was previously validated in pregnant women for each trimester [24]. All food items were automatically coded using the 2015 version of the Canadian Nutrient File [23] and data for energy and 22 nutrients were analyzed.

### 2.3. Supplement Use

Information regarding dietary and prenatal supplement use was obtained through a Web questionnaire administered within each trimester. Participants had to identify their supplement (e.g., brand name, type of supplement, specific nutrient, etc.), and provide its drug identification number (DIN), its measurement unit (e.g., tablets, drops, grams, milliliters, etc.), the dosage, and the frequency at which the reported dose was taken. Participants could enter as many as 10 dietary supplements. The Health Canada Licensed Natural Health Product Database [25] as well as companies’ product labels and websites were used to collect the nutritional information of all supplements entered by participants. If information was missing or was incomplete for any of the supplements’ characteristics, a research assistant contacted the participant to obtain the missing information. We assessed supplement use by compiling types of supplements used (multivitamins or single-nutrient supplements) and the number of users for each type of supplement.

### 2.4. Estimated Energy and Protein Requirements

Pre-pregnancy body weight was self-reported and height was measured at baseline to calculate pre-pregnancy BMI. Participants completed the validated French version of the Pregnancy Physical Activity Questionnaire (PPAQ) [26,27] within each trimester. Physical activity levels (PALs) were determined by ranking the participants according to the total amount of time they engaged in moderate and high-intensity activities (minutes/day). According to the Institute of Medicine (IOM) guidelines for the general adult population (which includes pregnant women) [3], participants were either considered sedentary (less than 30 min of moderate-intensity activity), low-active (30 to 60 min of moderate-intensity activity), active (60 to 180 min of moderate-intensity activity or 30 to 60 min of high-intensity activity) or very active (more than 180 min of moderate-intensity activity or more than 60 min of high-intensity activity). Estimated energy requirements (EERs) were calculated for each trimester by using pre-pregnancy weight, age, height, and physical activity coefficient corresponding to the PAL determined by the PPAQ [3]. An additional 340 kcal and 452 kcal were respectively added to the second and third trimester EERs [3]. Daily protein requirements were calculated as 1.1 g/kg of pre-pregnancy weight for the first 20 weeks of pregnancy, to which 25 g of protein per day was added for the remaining 20 weeks of pregnancy [3].

### 2.5. Other Variables

Gestational age (weeks of gestation) was confirmed by ultrasound conducted at the CHU de Québec—Université Laval in the first trimester. A Web-based self-administered questionnaire was completed by all participants either in the first (*n* = 62) or in the second (*n* = 24) trimester to collect information on economic and socio-demographic characteristics.

### 2.6. Statistical Analyses

within each trimester, means and standard deviations for energy and macro- and micronutrient intakes as well as the percentage of energy from carbohydrates (% carbohydrates), fat (% fat), and proteins (% proteins) were calculated from the three 24-h dietary recalls. Total micronutrient intakes were calculated by combining intakes from supplements and intakes from food sources only (derived from the dietary recalls). We then compared total energy and nutrient intakes and intakes from food sources only with dietary reference intakes (DRIs) by calculating proportions of women that had intakes below the estimated average intakes (EARs) and above the upper intake limit (UL), as applicable [28]. Folate intakes as dietary folate equivalent (DFE) were compared to the EAR (520 µg), and only synthetic forms of folic acid (i.e., fortified foods and supplements) were compared to the UL for folic acid (1000 µg), as the UL for folic acid applies only to synthetic forms [3]. Similarly, only niacin and magnesium intakes from supplements were compared to the UL for these nutrients, as their respective UL only applies to intakes from supplements [3]. Energy intakes (EIs) were compared with EERs, and protein, carbohydrate, and fat intakes as percentages of energy were compared with the acceptable macronutrient distribution range (AMDR) [3]. Proportions of women with values below or above the EERs or AMDR were calculated. Protein intakes (g/day) were also compared to estimated protein requirements, as previously described [3]. Finally, repeated measures ANOVA was performed to assess variations in energy, macro- and micronutrient intakes across trimesters. All statistical analyses were performed in JMP version 13 (SAS Institute Inc., Cary, NC, USA).

## 3. Results

Characteristics of the participants are presented in Table 1. Of the 86 pregnant women recruited, seven were lost to follow-up, mainly due to miscarriage or a lack of time to devote to the project. Therefore, results include 79 pregnant women with a mean age of 32.1 ± 3.7 years old and an average pre-pregnancy BMI of 25.7 ± 5.8 kg/m^2^. The majority of participants were Caucasian (97.5%), had a university degree (78.5%), an annual household income of C$80,000 or more (64.6%), and were multiparous (64.6%).

### 3.1. Supplement Use

Prenatal multivitamins were used by a majority of pregnant women (86.1%, 84.8%, and 78.5% in the first, second, and third trimesters, respectively) and folic acid supplements were the most commonly reported single-nutrient supplements (data not shown). Among women that did not take a multivitamin, the most reported single nutrient taken was folic acid for all trimesters (data not shown). Furthermore, among participants taking two supplements, the most reported single nutrients combined with a multivitamin were folic acid (50.0%) vitamin D (40.0%), and iron (44.4%) in the first, second, and third trimesters, respectively (data not shown). Small proportions (<10%) of women reported taking vitamin D, iron, and omega-3 as single-nutrient supplements throughout pregnancy (data not shown). In the third trimester, women who reported taking no supplement were significantly younger than the women who were taking at least one supplement (30.3 ± 3.8 years old vs. 32.6 ± 3.5 years old, *p* = 0.0236; data not shown).

### 3.2. Energy, Macronutrients, and Dietary Fiber

Table 2 shows trimester-specific energy intakes and macronutrient intakes as percentages of energy intake derived from the dietary recalls in comparison with EERs and AMDRs. No significant difference was observed for energy, protein, carbohydrate, or lipid intakes across trimesters. However, a significant increase in SFAs and a decrease in PUFAs as percentages of energy intakes were observed across trimesters (Table 2). Macronutrient intakes (grams per day) derived from the R24Ws and proportions of women that reported intakes above or below the corresponding DRIs are shown in Table 3. Mean energy intakes exceeded EERs in the first trimester (2294.3 ± 487.2 vs. 2122.4 ± 265.9 kcal; *p* = 0.006), but were below EERs in the third trimester (2234.6 ± 476.1 vs. 2492.2 ± 216.8 kcal; *p* < 0.0001). Protein intakes as a percentage of energy were within the acceptable distribution range (10–35%) in all trimesters but exceeded estimated requirements (1.1 g/kg) in the first trimester (96.7 ± 20.7 vs. 70.0 ± 8.6 g/day; *p* < 0.0001) for almost all participants (94.9% of them). In all trimesters, a majority of women reported fat intakes that were above the acceptable distribution range as a percentage of energy intakes. Inversely, carbohydrate intakes as percentages of energy were below the acceptable distribution range for more than 20% of participants for each trimester. Dietary fiber intakes were also below the DRI of 14 g/1000 kcal in all trimesters for more than 85% of participants.

### 3.3. Vitamins and Minerals

Micronutrient intakes derived from the R24Ws (food sources only) and proportions of women that reported intakes above or below the corresponding DRIs are shown in Table 4. A high prevalence of inadequate intakes was observed for vitamin D (93.7%, 83.5%, 78.5%), iron (88.6%, 89.9%, 94.9%), and folate (58.2%, 60.8%, 68.4%) in all trimesters, when only food sources were considered (Table 4). Vitamin B_6_ intakes were below the EAR for 36.7%, 32.9%, and 38.0% of women in the first, second, and third trimesters, respectively. Smaller proportions of women reported, throughout pregnancy, inadequate intakes of magnesium, vitamin A, calcium, and zinc. Vitamin C intakes were inadequate for 22.8% of participants in the second trimester but only for 4.1% and 10.1% of women in the first and third trimesters, respectively. Repeated measures ANOVA showed significant decreases in dietary intakes of vitamin C and manganese, as well as significant increases in dietary calcium and vitamin B_12_ intakes across trimesters (Table 4). In all trimesters, a majority of pregnant women reported sodium intakes that were above the UL of 2300 mg.

As shown in Table 5, when food sources and dietary supplements were combined, the proportion of women with adequate micronutrient intakes increased. With the exception of folate, vitamin D, and iron, less than 15% of our participants had total micronutrient intakes below the EAR, in all trimesters. Total intakes of folic acid and sodium were above the UL for a majority of women, and more than a third of participants had total iron intakes above the UL for all trimesters. The significant decrease observed for vitamin C and manganese, as well as the significant increase in calcium intakes persisted after the addition of intakes from supplements (Table 5).

## 4. Discussion

Our prospective assessment of pregnant women’s dietary intakes revealed a stability in energy and macronutrient intakes across trimesters. Most women exceeded their estimated requirements in terms of energy and protein in the first trimester but reported energy intakes below their needs later in pregnancy. We also found that diet alone may not be sufficient to provide adequate intakes for all micronutrients. Besides, when only food sources were considered, insufficient intakes of dietary fiber, vitamin D, folate, and iron were observed for a majority of women. Supplement use considerably improved the adequacy of micronutrient intakes among the pregnant women in our study sample, although excessive intakes were observed for iron, folic acid, and sodium.

Although it is recommended for pregnant women to increase their caloric intake as the pregnancy progresses [3], we found that there was a stability in energy intakes throughout pregnancy. Likewise, Abeysekera et al. [29] and Talai Rad et al. [30] as well as Moran et al. [18] found no significant changes in longitudinal caloric intakes of pregnant women. A prospective study by Vioque et al. conducted among Spanish pregnant women even observed a significant decrease in energy intakes (from the first to the third trimester) measured by a food frequency questionnaire (FFQ) [31]. Moreover, a recent meta-analysis of 18 studies by Jebeile et al. [32] reported little to no change in energy intake during pregnancy, which is in line with the stability we observed across trimesters. In light of their observations, Jebeile et al. [32] questioned the current caloric recommendations during pregnancy, suggesting they may be too high, but this affirmation should be further explored through studies that will focus on energy metabolism during pregnancy.

Although no variation in energy and macronutrient intakes was observed, most women exceeded their EER and EPR in the first trimester, in contrast with the third trimester, where a majority of women reported energy intakes below their EER. Kubota et al. [15] reported partially similar results, as they observed dietary intakes in the third trimester that were 900 kcal below the official Japanese recommendations. Since we do not have pre-pregnancy nutritional data, it is unknown whether the caloric excess observed is related to pregnancy itself or if it was already present before pregnancy. Augustine et al. [33] suggested that the process of «eating for two» associated with pregnancy occurs before the actual metabolic demand affects the mother. According to them, hormone-induced increase in dietary intakes early on in pregnancy could represent an adaptive response to the upcoming metabolic demand [33]. This could partially explain why our sample exceeded their EERs and EPRs as early as in the first trimester. The higher protein intakes observed in the first trimester also suggest that foods rich in protein (e.g., meat, dairy, legumes, etc.) may have contributed to the energy excess observed in early pregnancy, but this should be further investigated. Moreover, the questionnaire used to calculate PAL, the PPAQ, has been known to overestimate PAL in a small cohort of pregnant women [34]; therefore, the EERs calculated calculated within each trimester each trimester may have been overestimated. The use of a more precise method to measure our sample’s PAL (e.g., an accelerometer), could have attenuated the gap between EIs and EERs in the third trimester but could have increased it in the first trimester.

In parallel with the energy and protein excess observed in the first trimester, we found that, in all trimesters, more than half of our study sample reported fat intakes as percentages of energy that exceeded the acceptable range of 20–35%. These results are similar to those of Dubois et al. [11] in which a third of the 1533 pregnant women studied had total fat intakes as a percentage of energy above the recommended range. Furthermore, a meta-analysis by Blumfield et al. [19] also found that studies set in Western regions reported mean fat intakes (as percentages of energy intakes) of 35.0% to 37.1% among pregnant women, in accordance with our results. Moreover, in our study, 20.3% to 24.1% of participants reported carbohydrate intakes as a percentage of energy below the recommendations, which is also similar to other North American studies [19]. However, the literature is still incomplete and unclear on the roles that each macronutrient plays in pregnant women’s health [35]. Further research is therefore necessary to assess the impact of inadequate macronutrient intakes on maternal and fetal outcomes.

The suboptimal dietary intakes of fiber, vitamin D, folic acid, and iron observed in pregnant women from our study seem to be in line with the results of various authors [11,18,36,37,38,39]. Our results combined with those of other epidemiological studies thus suggest that food fortification policies and the use of a multivitamin during pregnancy are still necessary to reduce the risk of inadequate intake of micronutrients. In fact, our study showed that the use of dietary supplements greatly improved the adherence to micronutrient recommendations, as approximately 75% of all participants had total intakes above the EAR for all micronutrients. Dubois et al. [11] as well as Fayyaz et al. [40] reported similar results, especially regarding total iron and folate intakes. The insufficient intakes of dietary fiber observed in our study are in accordance with what Dubois et al. [11] reported, however, the relevance of these results and the impact of inadequate fiber intakes during pregnancy should be further investigated.

Most of our participants were supplement users and prenatal multivitamins were the most prevalent supplement taken by our study sample. It is important to mention that, although Health Canada recommends a prenatal multivitamin that contains 400 µg of folic acid and 16–20 mg of iron, close to all prenatal multivitamin supplements taken by our participants contained 1000 to 5000 µg of folic acid and 27 to 35 mg of iron (data not shown). Consequently, a majority of participants exceeded the UL for folic acid (1000 µg) in all trimesters and more than a third exceeded the UL for iron (45 mg) in the first and second trimesters. Dubois et al. [11] obtained similar results as they found that 90.4% and 32.4% of their participants had excessive folic acid and iron intakes, respectively, when dietary supplements were taken into account. Increased iron and folic acid intakes are indicated for women with conditions such as iron-deficiency anemia (iron) or for pregnant women at higher risk of giving birth to children with neural tube defects (folic acid) [41,42]. In our study, we do not have information regarding the number of women that were prescribed an iron supplement to prevent or to treat an iron-deficiency anemia. It is therefore impossible to know if the excessive iron intakes observed among our participants were due to anemia prevention or treatment. Moreover, it is important to mention that other nutrients, namely calcium, might decrease iron absorption, and thus observed total intakes of iron may not reflect the real iron status of our participants [43]. For these reasons, our results should be combined with direct assessment of iron status to evaluate the adequacy of our participant’s iron intakes. Furthermore, results from a recent Canadian study suggest that although fortification policies improved the population’s dietary intakes of folic acid, supplement users may be at risk of folic acid overconsumption [44]. To date, the implications of high folic acid intakes on pregnancy and prenatal health outcomes are not well understood and should therefore be further investigated [40,45,46].

Along with iron and folate, vitamin D was found to be one of the nutrients for which diet alone was insufficient to provide adequate intakes. The prevalence of inadequate intakes did decrease when dietary supplements were taken into account, but more than 20% of participants sill had total vitamin D intakes that were below the EAR, in all trimesters. Similar inadequacies were reported by Aghajafari et al. [37], as they found that 44% of their sample (*n* = 537 pregnant women) reported total vitamin D intakes (diet and supplements) that did not meet the Recommended Dietary Allowance (RDA) of 600 IU. Furthermore, despite the fact that more than half of their participants reported adequate daily vitamin D intakes (≥600 IU), Aghajafari et al. found that 20% of them were vitamin D-insufficient, according to the Endocrine Society and Osteoporosis Canada’s definition of 75 nmol/L circulating 25-hydroxyvitamin D. [47,48]. Moreover, Hollis et al. [49] conducted a double-blind randomized clinical trial in 494 pregnant women and found that a vitamin D supplementation of 4000 IU/day was the most effective in achieving vitamin D sufficiency, in comparison with 400 and 2000 IU/day. In our study, prenatal multivitamins taken by pregnant women contained, depending on the brand of the multivitamin, between 250 and 600 IU of vitamin D (data not shown). This may not be adequate, according to Hollis et al. [49], to complement dietary intakes of all pregnant women. Nevertheless, evidence regarding vitamin D supplementation during pregnancy is not currently sufficient to support definite clinical recommendations, and the results of Hollis et al. should be interpreted with caution [50]. It would also be necessary to combine dietary assessment (food and supplements) with direct measurement of vitamin D status (i.e., circulating 25(OH)D) and sun exposure in order to accurately evaluate vitamin D adequacy during pregnancy [11,51].

To our knowledge, this is the first study to prospectively assess whether or not pregnant women met current Canadian nutritional recommendations. The use of a validated Web-based 24 h recall combined with a Web questionnaire on supplement use generated detailed information on dietary and total intakes during pregnancy. However, our study has some limitations, namely regarding the small size and the lack of representativeness of our study sample, since most pregnant women enrolled were Caucasians and of a higher socioeconomic status. The nutritional inadequacies observed among our study sample may therefore be greater among less educated and lower-income pregnant women. Nutritional adequacy of pregnant women of lower socioeconomic status should be further investigated. However, despite our small sample size, our results highlight the need for more prospective, population-based studies regarding pregnant women’s dietary intakes, especially among lower income, less educated populations. Finally, our study did not measure circulating 25(OH)D in addition to iron and folate status, which limited our adequacy assessment of pregnant women’s vitamin D, iron, and folate intakes.

## 5. Conclusions

In summary, we observed that, contrary to current recommendations, there was a stability in dietary intakes across trimesters, and thus most women exceeded their energy and protein requirements in the first trimester and were had intakes below recommendations in the third trimester. The implications and possible causes of excessive energy and protein intakes in early pregnancy are not well documented and should be further investigated in association with gestational weight gain and other metabolic outcomes. The use of prenatal multivitamins and single nutrient supplements considerably improved iron, folate, and vitamin D adequacy, although excessive folic acid, iron, sodium, and niacin intakes were observed, and vitamin D inadequacies persisted for some pregnant women. Further research is needed to, firstly, evaluate the impact of high doses of folic acid on pregnancy and prenatal outcomes, and, secondly, to identify the dose of supplemental vitamin D necessary to achieve vitamin D sufficiency.

## Figures and Tables

**Table 1:** Participants’ characteristics (*n* = 79).

Variables	Mean ± SD or *N* (%)
Age (years)	32.1 ± 3.7
Weeks of gestation at baseline (weeks)	9.3 ± 0.7
Primiparous	28 (35.4)
BMI (kg/m^2^)	25.7 ± 5.8
Underweight	4 (5.1)
Normal weight	40 (50.6)
Overweight	20 (25.3)
Obese	15 (19.0)
Ethnicity–Caucasian	77 (97.5)
Education	
High school	4 (5.0)
College	13 (16.5)
University	62 (78.5)
Household income	
<C$40,000	5 (6.3)
C$40,000–59,999	10 (12.7)
C$60,000–79,999	12 (15.2)
C$80,000–99,999	18 (22.8)
>C$100,000	33 (41.8)
Income missing	1 (1.2)
Physical activity level (minutes of moderate and vigorous activity/day)	9.3 ± 0.7
First trimester	60.5 ± 59.6
Second trimester	45.9 ± 51.1
Third trimester	35.2 ± 41.5

**Table 2:** Trimester-specific energy intakes and macronutrient intakes as percentage of energy intakes in comparison with dietary reference intakes.

	First Trimester			Second Trimester			Third Trimester			
	Mean ± SD or AMDR Range	%Below AMDR or EER	%Above AMDR or EER	Mean ± SD or AMDR Range	%Below AMDR or EER	%Above AMDR or EER	Mean ± SD or AMDR Range	%Below AMDR or EER	%Above AMDR or EER	*p*-Value ^a^
EER (kcal/day)	2122.4 ± 265.9	-	-	2403.4 ± 241.1	-	-	2492.2 ± 216.8	-	-	
Energy intake (kcal/day)	2294.3 ± 487.2	36.7	63.3	2320.2 ± 519.1	60.8	39.2	2234.6 ± 476.1	70.9	29.1	0.09
AMDR protein, E%	10–35	-	-	10–35	-	-	10–35	-	-	-
Protein, E%	16.9 ± 2.5	0	0	17.3 ± 2.9	0	0	17.9 ± 3.3	0	0	0.14
AMDR carbohydrate, E%	45–65	-	-	45–65	-	-	45–65	-	-	-
Carbohydrate, E%	49.4 ± 4.7	20.3	0	48.3 ± 5.7	24.1	0	48.3 ± 5.5	24.1	0	0.27
AMDR total fat, E%	20–35	-	-	20–35	-	-	20–35	-	-	-
Total fat, E%	35.1 ± 4.0	0	50.6	35.8 ± 4.9	0	55.7	35.5 ± 4.4	0	57.0	0.53
SFA, E%	12.8 ± 2.1	-	-	13.2 ± 2.9	-	-	13.5 ± 2.5	-	-	0.047
MUFA, E%	12.3 ± 2.1	-	-	12.6 ± 2.0	-	-	12.5 ± 2.1	-	-	0.49
PUFA, E%	7.1 ± 1.9	-	-	7.1 ± 2.0	-	-	6.5 ± 1.8	-	-	0.03

^a^ *p*-value for repeated measures ANOVA performed to assess variations in energy and macronutrient intakes across trimesters. When no dietary reference intake was established for a nutrient, the “-” is used instead of a 0. AMDR: acceptable macronutrient distribution range; EER: estimated energy requirement, calculated with the following formula: 354 − (6.91 × age) + physical activity coefficient × [(9.36 × weight) + (726 × height)], to which an additional 340 or 452 kcal were added in the second and third trimesters. SFA: saturated fatty acids; MUFA: monounsaturated fatty acids; PUFA: polyunsaturated fatty acids.

**Table 3:** Trimester-specific macronutrient intakes in comparison with dietary reference intakes.

	First Trimester			Second Trimester			Third Trimester			
	Mean ± SD	%Below EPR or AI	%Above EPR or AI	Mean ± SD	%Below EPR or AI	%Above EPR or AI	Mean ± SD	%Below EPR or AI	%Above EPR or AI	*p*-Value ^a^
EPR, g/day	70.0 ± 8.6	-	-	95.0 ± 8.6	-	-	95.0 ± 8.6	-	-	-
Protein, g/day	96.7 ± 20.7	5.1	94.9	99.1 ± 20.9	48.1	51.9	98.2 ± 22.0	43.0	57.0	0.64
Carbohydrate, g/day	283.3 ± 68.8	-	-	280.3 ± 70.9	-	-	270.1 ± 68.0	-	-	0.14
Total fat, g/day	89.6 ± 21.7	-	-	93.1 ± 27.5	-	-	88.6 ± 23.6	-	-	0.20
Dietary fiber, g/day	23.3 ± 7.0	96.2	3.8	23.9 ± 8.0	87.3	12.7	22.9 ± 6.7	89.9	10.1	0.50
ω-6 Linoleic acid, g/day	14.8 ± 5.4	-	-	14.5 ± 5.3	-	-	13.4 ± 5.3	-	-	0.07
ω-3 Linolenic acid, g/day	2.0 ± 0.7	-	-	2.0 ± 0.9	-	-	1.9 ± 0.9	-	-	0.38
Cholesterol, mg/day	297.2 ± 99.7	-	-	291.1 ± 91.7	-	-	288.1 ± 109.0	-	-	0.81

^a^ *p*-value for repeated measures ANOVA performed to assess variations in macronutrient intakes across trimesters. When no dietary reference intake was established for a nutrient, the “-” is used instead of a 0. EPR: estimated protein requirement (1.1 g/kg or pre-pregnancy weight for the first half of pregnancy and 1.1 g/kg of pre-pregnancy weight + 25 g for the second half). AI: adequate intake.

**Table 4:** Trimester-specific micronutrient intakes from food alone in comparison with dietary reference intakes.

	EAR	UL	First Trimester			Second Trimester			Third Trimester			
			Mean ± SD	%Below EAR	%Above UL	Mean ± SD	%Below EAR	%Above UL	Mean ± SD	%Below EAR	%Above UL	*p*-Value ^a^
Vitamin D, IU/day	400	4000	234.8 ± 119.0	93.7	0	261.2 ± 135.2	83.5	0	271.9 ± 150.2	78.5	0	0.11
Iron, mg/day	22	45	15.3 ± 4.8	88.6	0	15.8 ± 5.2	89.9	0	14.8 ± 4.2	94.9	0	0.09
Folate, µg DFE/day	520	-	516.2 ± 139.5	58.2	-	495.4 ± 143.3	60.8	-	490.1 ± 141.3	68.4	-	0.31
Folic acid, µg/day	-	1000	155.0 ± 65.5	-	0	146.8 ± 72.6	-	0	138.3 ± 72.1	-	0	0.17
Vitamin B_6_, mg/day	1.6	100	1.8 ± 0.5	36.7	0	1.9 ± 0.5	32.9	0	1.8 ± 0.5	38.0	0	0.32
Magnesium, mg/day	290–300	350	381.2 ± 103.8	17.8	-	391.9 ± 108.9	19.0	-	386.2 ± 106.4	20.3	-	0.65
Vitamin A, µg RAE/day	550	3000	879.2 ± 305.9	13.9	0	906.4 ± 392.5	17.7	0	916.2 ± 398.4	17.7	0	0.71
Zinc, mg/day	9,5	40	12.5 ± 3.2	12.7	0	13.4 ± 3.2	8.9	0	13.2 ± 3.7	11.4	0	0.15
Calcium, mg/day	800	2500	1292.3 ± 381.8	10.1	0	1350.3 ± 515.9	13.9	2.5	1427.0 ± 506.0	6.3	1.3	0.02
Vitamin C, mg/day	70	2000	159.5 ± 66.7	5.1	0	137.9 ± 81.4	22.8	0	138.4 ± 66.6	10.1	0	0.01
Thiamin, mg/day	1.2	-	1.9 ± 0.6	5.1	-	1.9 ± 0.7	7.6	-	1.8 ± 0.8	7.6	-	0.40
Vitamin B_12_, µg/day	2.2	-	4.8 ± 1.6	2.5	-	5.4 ± 2.4	3.8	-	5.6 ± 2.5	0	-	0.02
Riboflavin, mg/day	1.2	-	2.3 ± 0.6	1.3	-	2.4 ± 0.6	1.3	-	2.5 ± 0.8	0	-	0.07
Niacin, mg NE/day	14	35	45.7 ± 10.6	0	-	45.9 ± 9.2	0	-	45.0 ± 10.3	0	-	0.64
Pantothenic acid, mg/day	-	-	6.5 ± 1.8	-	-	6.5 ± 1.5	-	-	6.5 ± 1.6	-	-	0.98
Phosphorus, mg/day	580	3500	1616.4 ± 383.7	0	0	1660.3 ± 398.0	0	0	1673.7 ± 442.1	0	0	0.47
Sodium, mg/day	-	2300	3406.0 ± 889.8	-	94.9	3276.0 ± 950.3	-	86.1	3199.0 ± 921.7	-	84.8	0.17
Manganese, mg/day	-	11	4.0 ± 1.5	0	1.3	4.3 ± 1.4	-	0	3.9 ± 1.3	-	0	0.005
Selenium, µg/day	49	400	135.7 ± 34.0	0	0	135.2 ± 32.1	0	0	131.8 ± 30.9	0	0	0.48
Copper, mg/day	0.8	10	1.5 ± 0.6	1.3	0	1.6 ± 0.6	1.3	0	1.5 ± 0.5	3.8	0	0.22

^a^ *p*-value for repeated measures ANOVA performed to assess variations in micronutrient intakes across trimesters. When no EAR or UL was established for a nutrient, the “-” is used instead of a 0. EAR: estimated average requirement; UL: upper intake limit; DFE: dietary folate equivalent; RAE: retinol activity equivalents; NE: niacin equivalent.

**Table 5:** Trimester-specific total micronutrient intakes (including food sources and supplements) in comparison with dietary reference intakes.

	EAR	UL	First Trimester			Second Trimester			Third Trimester			*p*-Value ^a^
			Mean ± SD	%Below EAR	%Above UL	Mean ± SD	%Below EAR	%Above UL	Mean ± SD	%Below EAR	%Above UL	
Vitamin D, IU/day	400	4000	632.2 ± 555.9	25.3	1.3	690.0 ± 538.4	21.5	1.3	689.4 ± 544.9	21.5	1.3	0.15
Iron, mg/day	22	45	38.2 ± 14.0	19.0	35.4	38.8 ± 13.8	19.0	38.0	41.0 ± 24.7	22.8	36.7	0.65
Folate, µg DFE/day	520	-	1777.0 ± 1221.4	7.6	-	1763.2 ± 1313.0	10.1	-	1617.6 ± 1212.2	16.5	-	0.29
Folic acid, µg/day	-	1000	1415.8 ± 1213.7	-	86.1	1412.6 ± 1313.4	-	83.5	1265.8 ± 1199.3	-	79.7	0.27
Vitamin B_6_, mg/day	1.6	100	5.6 ± 3.9	8.9	0	5.8 ± 4.0	7.6	0	5.4 ± 4.0	8.9	0	0.29
Magnesium, mg/day	290–300	350	419.1 ± 108.4	8.9	0	431.9 ± 113.9	8.9	0	424.2 ± 110.8	11.4	0	0.62
Vitamin A, µg RAE/day	550	3000	1398.1 ± 574.4	7.6	0	1415.9 ± 674.8	7.6	0	1429.8 ± 635.5	7.6	0	0.91
Zinc, mg/day	9,5	40	20.4 ± 6.7	5.1	0	21.4 ± 6.6	2.5	0	21.0 ± 7.2	3.8	0	0.36
Calcium, mg/day	800	2500	1503.4 ± 370.6	2.5	0	1560.0 ± 551.4	6.3	5.1	1630.7 ± 524.4	2.5	6.3	0.04
Vitamin C, mg/day	70	2000	234.7 ± 79.1	1.3	0	215.7 ± 93.3	7.6	0	213.2 ± 71.0	2.5	0	0.04
Thiamin, mg/day	1.2	-	3.4 ± 1.3	2.5	-	3.5 ± 1.3	2.5	-	3.3 ± 1.4	2.5	-	0.31
Vitamin B_12_, µg/day	2.2	-	11.0 ± 8.0	1.3	-	10.6 ± 5.7	0	-	10.1 ± 5.3	0	-	0.54
Riboflavin, mg/day	1.2	-	4.0 ± 1.4	1.3	-	4.1 ± 1.5	0	-	4.1 ± 1.5	0	-	0.80
Niacin, mg NE/day	14	35	60.1 ± 13.4	0	-	60.8 ± 12.8	0	-	59.3 ± 14.6	0	-	0.64
Pantothenic acid, mg/day	-	-	11.1 ± 3.2	-	-	11.1 ± 3.1	-	-	10.9 ± 3.1	-	-	0.86
Phosphorus, mg/day	580	3500	1618.0 ± 383.6	0	0	1665.7 ± 401.2	0	0	1673.7 ± 442.1	0	0	0.49
Sodium, mg/day	-	2300	3406.0 ± 889.8	-	94.9	3276.0 ± 950.3	-	86.1	3199.0 ± 921.7	-	84.8	0.17
Manganese, mg/day	-	11	5.0 ± 1.9	-	1.3	5.3 ± 1.8	-	1.3	4.9 ± 1.6	-	0	0.04
Selenium, µg/day	49	400	151.2 ± 38.1	0	0	151.4 ± 39.2	0	0	147.6 ± 37.5	0	0	0.57
Copper, mg/day	0.8	10	2.6 ± 0.9	1.3	0	2.7 ± 1.0	1.3	0	2.5 ± 0.9	2.5	0	0.23

^a^ *p*-value for repeated measures ANOVA performed to assess variations in micronutrient intakes across trimesters. When no EAR or UL was established for a nutrient, the “-” is used instead of a 0. EAR: estimated average requirement; UL: upper intake limit; DFE: dietary folate equivalent; RAE: retinol activity equivalents; NE: niacin equivalent.
