# Correction: Savard et al. Trimester-Specific Dietary Intakes in a Sample of French-Canadian Pregnant Women in Comparison with National Nutritional Guidelines. *Nutrients* 2018, *10*, 768

**DOI:** 10.3390/nu11010084

**Published:** 2019-01-04

**Authors:** Claudia Savard, Simone Lemieux, S. John Weisnagel, Bénédicte Fontaine-Bisson, Claudia Gagnon, Julie Robitaille, Anne-Sophie Morisset

**Affiliations:** 1 School of Nutrition, Laval University, Québec City, QC G1V 0A6, Canada; claudia.savard.4@ulaval.ca (C.S.); simone.lemieux@fsaa.ulaval.ca (S.L.); julie.robitaille@fsaa.ulaval.ca (J.R.); 2 Endocrinology and Nephrology Unit, CHU de Québec-Université Laval Research Center, Québec City, QC G1V 4G2, Canada; john.weisnagel@crchudequebec.ulaval.ca (S.J.W.); claudia.gagnon@crchudequebec.ulaval.ca (C.G.); 3 Institute of Nutrition and Functional Foods, Laval University, Québec City, QC G1V 0A6, Canada; 4 Department of Medicine, Laval University, Québec City, QC G1V 0A6, Canada; 5 School of Nutrition Sciences, University of Ottawa, Ottawa, ON K1N 6N5, Canada; bfontain@uottawa.ca; 6 Institut du Savoir Montfort, Montfort Hospital, Ottawa, ON K1K 0T2, Canada

The authors wish to make the following changes to their paper (Savard et al., 2018 [1]). While re-analyzing our data for a new manuscript (using the same database), we observed that some of our participants’ characteristics were different compared to those presented in our previously published work. The mistakes were unintentional and do not impact the main findings of our paper, as they were only related to descriptive analyses (see below: Table 1; data on pre-pregnancy body mass index (BMI) and household income).

**Published version:**

**Table 1:** Participants’ characteristics (*n* = 79).

Variables	Mean ± SD or *N* (%)
Age (years)	32.1 ± 3.7
Weeks of gestation at baseline (weeks)	9.3 ± 0.7
Primiparous	28 (35.4)
BMI (kg/m^2^)	25.7 ± 5.8
Underweight	4 (5.1)
Normal weight	40 (50.6)
Overweight	20 (25.3)
Obese	15 (19.0)
Ethnicity–Caucasian	77 (97.5)
Education	
High school	4 (5.0)
College	13 (16.5)
University	62 (78.5)
Household income	
<C$40,000	5 (6.3)
C$40,000–59,999	10 (12.7)
C$60,000–79,999	12 (15.2)
C$80,000–99,999	18 (22.8)
>C$100,000	33 (41.8)
Income missing	1 (1.2)
Physical activity level (minutes of moderate and vigorous activity/day)	9.3 ± 0.7
First trimester	60.5 ± 59.6
Second trimester	45.9 ± 51.1
Third trimester	35.2 ± 41.5

Results section, page 4/14 (as published):

**3. Results**

Characteristics of the participants are presented in Table 1. Of the 86 pregnant women recruited, seven were lost to follow-up, mainly due to miscarriage or lack of time to devote to the project. Therefore, results include 79 pregnant women with a mean age of 32.1 ± 3.7 years and an average pre-pregnancy BMI of 25.7 ± 5.8 kg/m^2^. The majority of participants were Caucasian (97.5%), had a university degree (78.5%), an annual household income of C$80,000 or more (64.6%), and were multiparous (64.6%).

**Revised version:**

**Table 1:** Participants’ characteristics (*n* = 79).

Variables	Mean ± SD or *N* (%)
Age (years)	32.1 ± 3.7
Weeks of gestation at baseline (weeks)	9.3 ± 0.7
Primiparous	28 (35.4)
BMI (kg/m^2^)	25.7 ± 5.8
Underweight	2 (2.5)
Normal weight	43 (54.4)
Overweight	19 (24.1)
Obese	15 (19.0)
Ethnicity–Caucasian	77 (97.5)
Education	
High school	4 (5.0)
College	13 (16.5)
University	62 (78.5)
Household income	
<C$40,000	5 (6.3)
C$40,000–59,999	10 (12.7)
C$60,000–79,999	13 (16.5)
C$80,000–99,999	17 (21.5)
>C$100,000	33 (41.8)
Income missing	1 (1.2)
Physical activity level (minutes of moderate and vigorous activity/day)	
First trimester	60.5 ± 59.6
Second trimester	45.9 ± 51.1
Third trimester	35.2 ± 41.5

Results section, page 4/14 (revised version):

**3. Results**

Participant characteristics are presented in Table 1. Of the 86 pregnant women recruited, seven were lost to follow-up, mainly due to miscarriage or lack of time to devote to the project. Therefore, results include 79 pregnant women with a mean age of 32.1 ± 3.7 years and an average pre-pregnancy BMI of 25.7 ± 5.8 kg/m^2^. The majority of participants were Caucasian (97.5%), had a university degree (78.5%), an annual household income of C$80,000 or more (63.3%), and were multiparous (64.6%).

The authors would like to apologize for any inconvenience caused to the readers by this change. The changes do not affect the results and we apologize for the oversight.
